# Old drugs, new tricks: leveraging known compounds to disrupt coronavirus-induced cytokine storm

**DOI:** 10.1038/s41540-022-00250-9

**Published:** 2022-10-10

**Authors:** Spencer Richman, Cole Lyman, Anastasia Nesterova, Anton Yuryev, Matthew Morris, Hongbao Cao, Chris Cheadle, Gary Skuse, Gordon Broderick

**Affiliations:** 1 Rochester General Hospital, Center for Clinical Systems Biology, Rochester, NY USA; 2 Elsevier BV, Biology Solutions, Amsterdam, the Netherlands; 3 Rochester Institute of Technology, Gosnell School of Life Sciences, Rochester, NY USA

**Keywords:** Virtual drug screening, Molecular medicine

## Abstract

A major complication in COVID-19 infection consists in the onset of acute respiratory distress fueled by a dysregulation of the host immune network that leads to a run-away cytokine storm. Here, we present an in silico approach that captures the host immune system’s complex regulatory dynamics, allowing us to identify and rank candidate drugs and drug pairs that engage with minimal subsets of immune mediators such that their downstream interactions effectively disrupt the signaling cascades driving cytokine storm. Drug–target regulatory interactions are extracted from peer-reviewed literature using automated text-mining for over 5000 compounds associated with COVID-induced cytokine storm and elements of the underlying biology. The targets and mode of action of each compound, as well as combinations of compounds, were scored against their functional alignment with sets of competing model-predicted optimal intervention strategies, as well as the availability of like-acting compounds and known off-target effects. Top-ranking individual compounds identified included a number of known immune suppressors such as calcineurin and mTOR inhibitors as well as compounds less frequently associated for their immune-modulatory effects, including antimicrobials, statins, and cholinergic agonists. Pairwise combinations of drugs targeting distinct biological pathways tended to perform significantly better than single drugs with dexamethasone emerging as a frequent high-ranking companion. While these predicted drug combinations aim to disrupt COVID-induced acute respiratory distress syndrome, the approach itself can be applied more broadly to other diseases and may provide a standard tool for drug discovery initiatives in evaluating alternative targets and repurposing approved drugs.

## Introduction

Though statistics vary between sites and are dependent on a host of comorbidities, between 20 and 60% of COVID-19 infected patients admitted to hospital develop acute respiratory distress syndrome (ARDS)^1,2^, with complications being a primary contributor to the mortality in up to 50% of these instances. Indeed, early evidence has suggested that COVID-19-induced ARDS may represent a specific variant thereof^3,4^. ARDS arises from complex interactions between components of the immune inflammatory response^5,6^ including complement activation^7^. This underlying inflammatory response appears especially aggressive in many cases of COVID-19 infection, with the self-aggravating release of increasing amounts of inflammatory cytokines fueling a run-away cytokine storm^8,9^. Though antiviral and immune-modulating agents are undergoing clinical trial, the sudden emergence and rapid global spread of COVID-19 has highlighted the quintessential value of rapidly identifying and redirecting tried and true pharmaceutical agents to immediately mitigate illness severity. This being said, the recent ad hoc use and subsequent failure of the antimalarial hydroxychloroquine in formal clinical trials^10^ suggests that important challenges remain if this is to be accomplished safely, effectively, and reliably. Current efforts have for the most part been focused on eliminating the root cause of infection with significant resources being applied to the characterization of SARS-CoV-2 molecular targets and the identification of novel antiviral compounds using molecular docking as the primary computational workhorse^11,12^. While conventional analyses of this type typically focus directly on individual virally encoded proteins, recent efforts have attempted to broaden their scope by mapping the pathogen-host interactome to identify downstream targets in the viral replication machinery that might be readily modulated using known compounds^13,14^. Though vital, these efforts are, by virtue of their single molecule resolution, both experimentally and computationally resource-intensive and time-consuming. An alternative and highly complementary means of reducing mortality in COVID-induced ARDS consist in appropriately modulating host immune response such that cytokine storm is averted. Unfortunately, while the molecular affinity approaches mentioned above are well-suited for identifying drugs binding with high specificity to a given target they require prior knowledge of what the appropriate target should be. Moreover, this time-independent analysis of binding affinity is ill-suited to describe the complex dynamics of immune cell signaling that lead to persistent cytokine storm where regulatory kinetics play a vital role in the selection of therapeutic targets.

Here, we propose a novel and expedient in silico approach for the evaluation and selection of drug repurposing candidates directed at model-predicted targets that formally account for the regulatory dynamics of the network as a whole. Utilizing both experimental and model-predicted affinities as well as literature-mined drug–target interaction data, we assemble and score multidrug therapies based on their expected regulatory actions on sets of concurrent molecular targets that act synergistically to disrupt cytokine storm in the context of coronavirus infection. Issues with data sparsity which are of special relevance in sudden and rapidly evolving health crises such as the COVID-19 pandemic, undermine the reliable use of data-driven approaches but can be circumvented by maximally leveraging the extraction of currently existing knowledge from the peer-reviewed literature. In previous work by our group^15^, we extracted prior knowledge from 2653 peer-reviewed publications to construct a network that included 18 host immune mediators documented to be of specific relevance to coronavirus infection, the latter being annotated as a disease entity in the Elsevier Knowledge Graph text-mining database^16^. These 18 immune mediators and the coronavirus pathogen were linked by 112 documented regulatory actions to create a network model that was capable of supporting biologically plausible immune response dynamics that included a persistent cytokine storm cascade^17^. Often lethal once triggered, this excessive and self-perpetuating host inflammatory response may persist in the absence of a pathogen as demonstrated recently in ref. ^9^. The objective of the current work therefore is to use the above-mentioned network model of host immune response to coronavirus infection as a basis for predicting intervention strategies capable of disrupting this run-away inflammatory cascade. Toward this, we conduct simulations of the host inflammatory response as part of an optimal search for companion drugs that would optimally compliment the actions of a concurrently administered antiviral, simulated here as an idealized reduction in viral titer. We focus in this first analysis on FDA-approved drugs as they might be more readily deployed but more importantly because there exists a broader more established base of clinical experience regarding their effects, compared to that of experimental compounds.

Our simulations highlighted several drugs and drug combinations that show promise in disrupting cytokine storm when administered as companions to an effective antiviral. In addition to recovering, known immunosuppressants like rapamycin and cyclosporine, as well as the corticosteroid dexamethasone, model predictions also correctly assigned a low score to hydroxychloroquine and chloroquine, two early contenders later proven to be much less effective than anticipated. Most importantly perhaps, our analysis pointed to several highly novel opportunities for significantly enhancing the actions of dexamethasone by pairing the latter with a select list of drugs that include antimicrobials such as ciprofloxacin or levofloxacin, and the statin simvastatin. Though these remain predictions, we propose that this model-based framework, because it is rooted in a broad body of prior knowledge, can be highly useful in focusing attention to drug candidates with significant potential and do so quickly with minimal data.

## Results

### Idealized interventions and suitability for repurposing

**Fig. 1:**
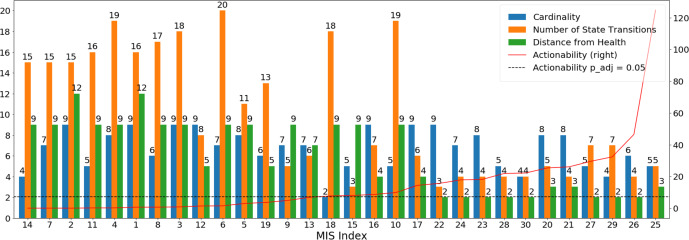
Suitability of optimal target sets for drug repurposing. A quantitative ranking of 30 model-predicted idealized interventions of various cardinality (green bars), length of response trajectory or efficiency (orange bars) and distance from optimal outcome (blue bars) as a function of their actionability (red line) or potential for translation into clinic using repurposing of currently available drugs. Only 16 of the initial 30 solution sets are significantly more accessible (empirical null distribution *P* < 0.05; A > A_crit, *P*=0.05_ ~7.25) to repurposing than a random MIS solution set (dashed blue line).

Of the 19 original network models^15^, we focus here specifically on model 18, which we believe supports the most biologically plausible predicted immune response dynamics based on criteria described in^17^. Stating the search for idealized intervention sets as a constraint satisfaction problem^18^, we identified 30 intervention solutions^15^ that prompt immune signaling predicted by the network model to migrate from a pattern of persistent immune hyperactivation (cytokine storm) to one that more closely resembles an idealized immune resting state. These solutions were computed under conditions of low viral load to highlight best case performance and because they would be used in concert with an effective antiviral treatment. The exact combinations of exogenous manipulations proposed in each of these theoretically optimal solutions *s*_*i*_ are listed in Supplementary Table 1 (Additional File 1) along with summary measures of optimality, namely the number of exogenous manipulations, the outcome residual distance from the target immune resting state, the number of transitions required to achieve this outcome as well as the overall pharmacologic actionability A(*s*) as it applies to the repurposing of the 144 candidate drugs. The latter is reported along with the significance of this value compared to that of a random intervention set, adjusted for multiple comparisons (*P*_adj_). These summary measures are also presented graphically in Fig. 1. Results show that 16 of the 30 optimal intervention sets score as significantly actionable (empirical null distribution *P* < 0.05; A > 7.25) in the 144 candidate drugs compared to a random intervention set. Interestingly these 16 significantly actionable solutions involve only slightly fewer immune targets on average (~6 targets) than their nonsignificant counterparts (~7 targets) despite this being a component of the actionability score. This similarity notwithstanding, the specific combinations of targets selected in each of these 16 solutions effectively exploit the regulatory network structure in a way that supports a much more expedient or efficient migration (~7 versus ~14 transitions) to outcome states where at least 85% of immune marker expression is restored to the baseline healthy resting state (~4 versus ~9 activation increments from an initial separation of 27). Of the 18 immune proteins in the network, TNF, IFNL1 (aka IL-29), and CSF3 are almost unanimously shared among the most actionable solutions. Indeed, IFNL1 and TNF appear in almost twice as many significantly actionable MIS solutions as they do in their nonsignificant counterparts. The two most actionable idealized interventions both consist of a joint inhibition of CSF3, CTSL, CXCL2, IFNG, and TNF, with one also inhibiting IFNL1. Interestingly recent work by^9^ supports a central role for type II interferon IFNG and TNF synergy in COVID-related cytokine shock. However, much less is known about a direct role of type III IFNL1 in a cytokine storm, though recent work proposes the latter as a major mediator of thromboinflammation^19^, an emerging clinical feature of COVID-19 infection^20^.

### Ranking individual drugs

**Fig. 2:**
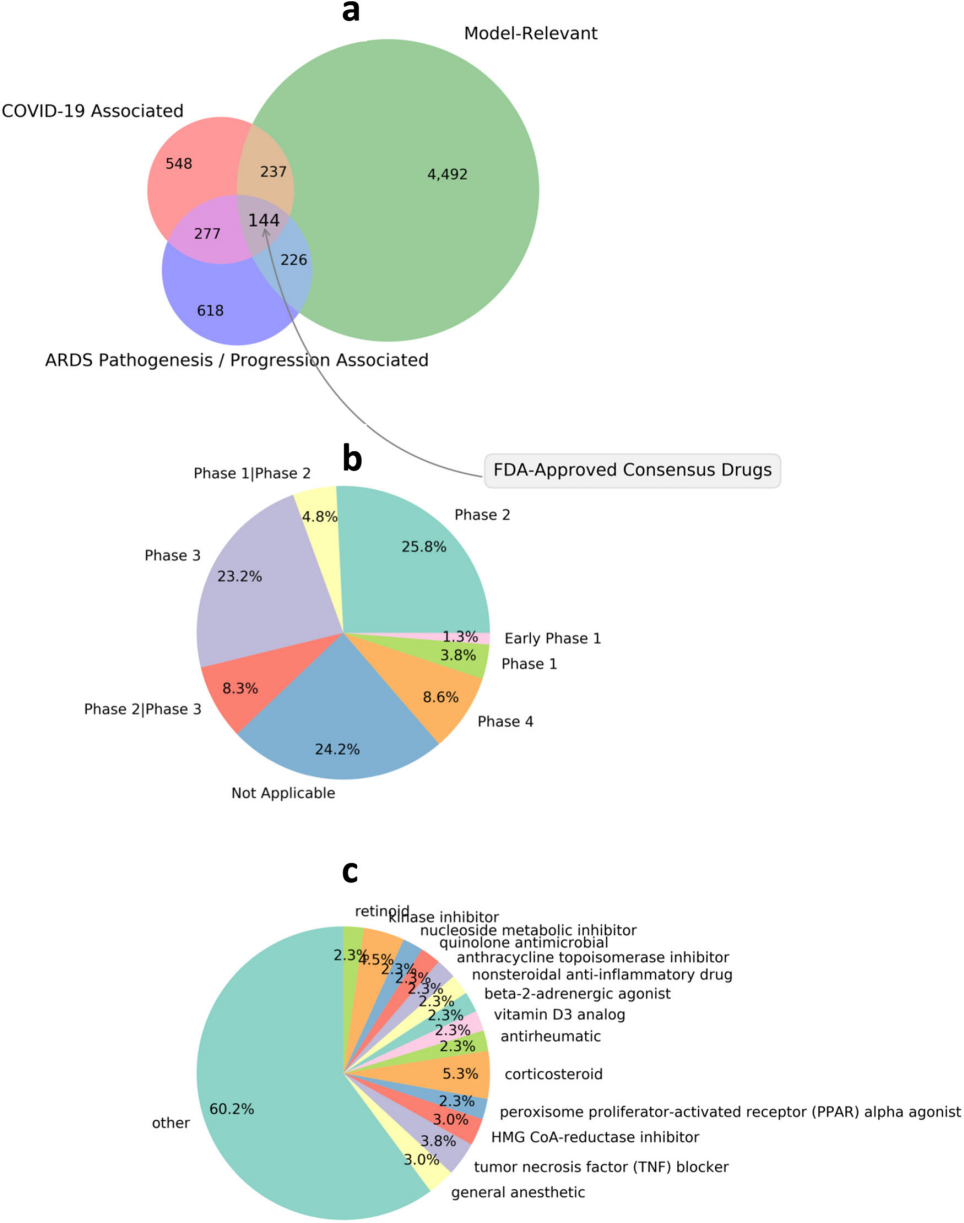
A multi-pronged search for candidate compounds. **a** A total of 144 FDA-approved drugs were identified with consensus between three independent search methodologies. **b** Breakdown of the status of the subset of 67 drugs currently registered to clinical trials (July 27, 2020). **c** Breakdown of the 144 consensus drugs into the subset of 94 FDA Established Pharmacologic Classes (EPC) (of 540 EPC classes). Classes with fewer than three instances were collapsed to the other category.

In order to identify drug repurposing strategies that might be translatable into clinical practice, we computed for each drug and drug combination the adjusted enrichment *E*_adj_ in each of the 16 significantly actionable intervention sets. Of the 144 FDA-approved candidate drugs that were identified as illness and model-relevant (Fig. 2a), no single drug scored a weighted adjusted median enrichment (WM *E*_adj_) that significantly exceeded that of a random selection from the background set of model relevant drugs in even one idealized MIS solution (all *P*_adj_ (max *E*_adj_) > 0.10) (Additional File 1, Supplementary Table 2). Indeed, only eight drugs scored above 0.30, with dexamethasone being among these and rapamycin ranking highest with a WM *E*_adj_ of 0.37. The much-publicized hydroxychloroquine (CQ) and chloroquine (CQ) aligned very poorly with model-predicted intervention sets, delivering WM *E*_adj_ values of 0.13 and 0.05, respectively, and at best a maximal *E*_adj_ of 0.34 and 35 in any single solution. The highest adjusted enrichment produced in any single MIS (max *E*_adj_) was delivered by the progestin medroxyprogesterone acetate, typically associated with menopausal hormone therapy but whose immune-modulatory actions have been highlighted recently^21^. Recall that the 16 significantly actionable interventions called for 6 targets on average to be manipulated (Additional File 1, Supplementary Table 1), a number consistent with the broad actions of drugs such as rapamycin, documented to inhibit eight network targets, as well as medroxyprogesterone acetate and dexamethasone, each known to inhibit six and nine network targets, respectively. However, this broad coverage of targets come at a price. Indeed, penalties applied to control for direct off-target effects as well as drug actions contrary to those prescribed by the MIS solutions were such that drugs like the antimicrobial ciprofloxacin, which is documented to inhibit only three network targets, may nonetheless produce enrichment scores similar to that of dexamethasone. Though very different in their breadth of coverage, both dexamethasone and ciprofloxacin were among the least generalizable across MIS solutions with median absolute deviations (0.16; 0.17, respectively) equivalent to half their weighted median *E*_adj_ performance (0.32; 0.34, respectively). In contrast, medroxyprogesterone acetate would appear to target network elements that are better conserved across intervention solutions with a median absolute deviation of 0.05 for a weighted median performance of 0.37, suggesting the latter more effectively targets complementary intervention pathways and may be more robust to model uncertainty. A similar argument could be made for the use of the calcineurin inhibitor cyclosporine.

**Fig. 3:**
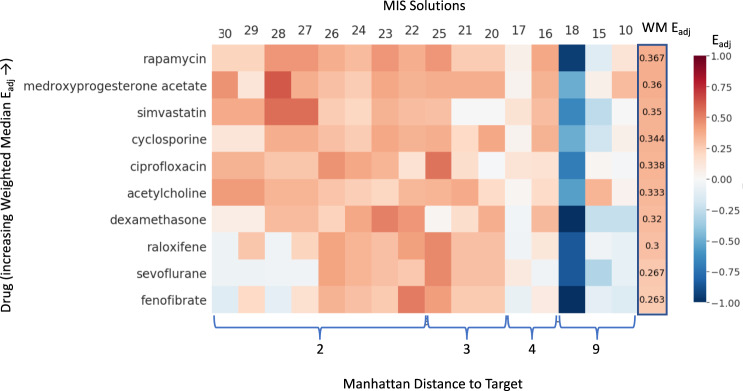
Ranking single drugs. Adjusted enrichment *E*_adj_ values obtained in each of the 16 significantly actionable MIS interventions (empirical null distribution p(*A(s)*) < 0.05) computed for the individual drugs with the top ten highest overall weighted median adjusted enrichment (WM *E*_adj_). Drugs are ordered along the vertical axis by increasing weighted median *E*_adj_ and MIS solutions along the horizontal axis by increasing residual Manhattan distance (bits) separating the treatment outcome from the target healthy resting state.

The variability in performance across individual intervention solutions is further illustrated in Fig. 3. As expected, drugs at the top of the heatmap like rapamycin offer more consistent performance across those MIS solutions where treatment outcomes deviate by only two or three bits from the desired full- recovery profile. Indeed, even though drugs like raloxifene, sevoflurane (e.g., solution 25) and fenofibrate (e.g., solution 22) achieve higher adjusted enrichment scores in some isolated instances, they also present with no or even negative enrichment values in multiple other intervention solutions giving them a lower overall ranking. While most intervention solutions were supported reasonably well by at least one drug, this was not the case for solution 18 where all top-ranking drugs performed very poorly, presenting with uniformly negative enrichment scores. Intervention 18 (Additional File 1, Supplementary Table 1) is the single-most parsimonious of all significantly actionable intervention sets, consisting of only two-target manipulations, namely a downregulation of CD86 conducted in unison with an upregulation of TNF. As all other intervention sets involve four or more targets, broader-acting drugs are being favored overall, leading to a large overenrichment penalty for a two-target solution like that proposed under MIS 18. Of note, the importance of introducing drugs that more adequately align with solution 18 is undermined further by the latter’s poor outcome which is among the 3 worst recovery profiles predicted (i.e., 9 bits separation from full recovery).

### Ranking drug combinations

**Fig. 4:**
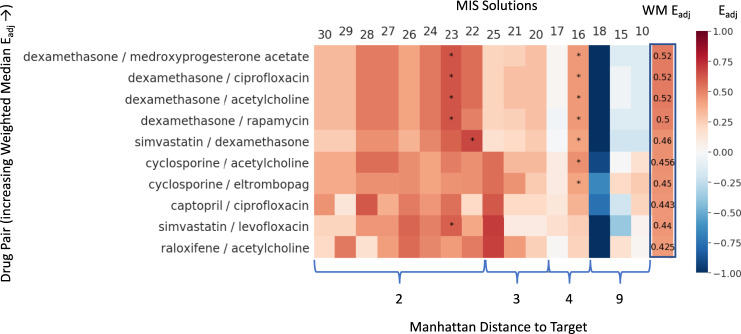
Ranking drug combinations. Adjusted enrichment *E*_adj_ values obtained in each of the 16 significantly actionable MIS interventions (empirical null distribution p(*A(s)*) < 0.05) computed for the drug pairs with the top ten highest overall weighted median adjusted enrichment (WM *E*_adj_). Drug pairs are ordered along the vertical axis by increasing weighted median *E*_adj_ and MIS solutions along the horizontal axis by increasing residual Manhattan distance (bits) separating the treatment outcome from the target healthy resting state. Asterisks indicate an *E*_adj_ value with an empirical null distribution p(*E*_adj_) < 0.05 significance.

Of the roughly 10,000 possible pairwise combinations of the 144 candidate drugs, only 9 such pairs were enriched in at least one MIS solution at a significance of *P*_adj_(Max *E*_adj_) < 0.05, with the corresponding significance in weighted median adjusted enrichment *P*_adj_(WM *E*_adj_) < 0.10, both based on an empirical null distribution (Additional File 1, Supplementary Table 3). With a weighted median *E*_adj_ ranging from 0.46 to 0.52, all five significant drug pairs consisted of combining the corticosteroid dexamethasone with one of several companion drugs, namely cholinergic agonist acetylcholine, the progestin medroxyprogesterone acetate, the antimicrobial ciprofloxacin, the mTOR inhibitor rapamycin or the statin simvastatin. Offering a slightly lower weighted median *E*_adj_ of 0.45 but with a much more consistent performance across MIS solutions was the combination of immunosuppressant cyclosporine with eltrombopag, a drug typically used to treat thrombocytopenia or abnormally low platelet count. Other high-ranking drug pairs also include the antimicrobial levofloxacin, the ACE inhibitor captopril, and the selective estrogen receptor modulator (SERM) raloxifene. Here again, the performance of the top ten drug pairs at the level of individual intervention solutions is illustrated as a heatmap in Fig. 4. In contrast with the results presented in Fig. 3, we find strictly positive-enrichment scores for all drug pairs in intervention solutions with outcomes 2 to 3 bits away from a full recovery profile. Indeed, we find broad swaths of high positive-enrichment scores across the MIS solutions with the best outcomes (i.e., two bits separation) in particular for the top five drug pairs. Conversely, the added target coverage typically achieved by a drug pair only further exacerbates the overenrichment of MIS solution 18 leading to even more negative enrichment scores.

**Fig. 5:**
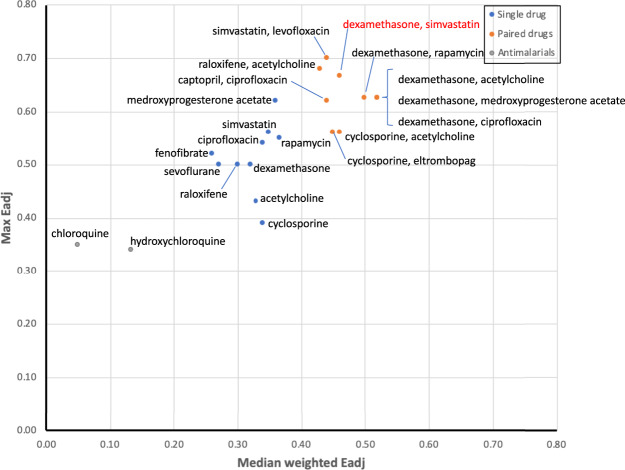
Broad vs focused performance. Maximum adjusted enrichment (max *E*_adj_) obtained for any single idealized intervention (MIS) versus the aggregate performance expressed as the weighted median adjusted enrichment (WM *E*_adj_) across all 16 significantly actionable MIS solutions for the top ten ranked single drugs (blue dots) and drug pairs (orange dots) (Additional File 1, Supplementary Tables 2 and 3).

Ultimately, we would like a drug or drug pair to show the highest possible enrichment *E*_adj_ in at least one intervention solution (Max *E*_adj_) and have it maintain this enrichment across as many of the best performing MIS solutions as possible, i.e., also show a high weighted median *E*_adj_. Results presented in Fig. 5 show that most top-ranking drug pairs typically deliver a maximum enrichment *E*_adj_ equivalent or superior to that of single drugs. Moreover, all such pairs invariably maintain this performance more readily across those alternative MIS solutions producing more desirable outcomes. Indeed, while the combination of dexamethasone and medroxyprogesterone acetate offers only a slight improvement in maximum enrichment over medroxyprogesterone acetate alone (max *E*_adj_ 0.63 over 0.62), this improvement is much better sustained across those alternative intervention solutions that correspond to more favorable outcomes (WM *E*_adj_ of 0.52 over 0.36). Introducing simvastatin in combination with dexamethasone or levofloxacin offers the potential for even higher maximum enrichments of 0.67 and 0.70, respectively, albeit with moderate reductions in general applicability across intervention paths (WM *E*_adj_ 0.46 and 0.44, respectively, from 0.52). In contrast, combining simvastatin with the JAK1/2 kinase inhibitor ruxolitinib, a combination currently in Phase 2 trials (NCT04348695), scored poorly in both metrics and ranked 662nd among all combinations. Generally, one can reason that combining drugs that offer highly complementary drug actions will increase their target coverage and more readily support a broader range of alternative intervention solutions. Conversely, drug pairs that offer higher maximum performance but do so on a narrower range of solutions might be expected to perform more like single drugs as their actions overlap, limiting their ability to support alternative treatment paths.

Combination therapies offer the potential of broader overall drug action, however, they also carry the possibility for negative drug-drug interactions. While severity rating categories differ from one drug information resource to another^22^, recently proposed a standardized severity scale that reconciled definitions across multiple sources into three basic severity categories, namely Major, Moderate and Mild severity. In this work, we extracted drug interaction severities for our top-ranking drug pairs (Additional File 1, Supplementary Table 3) as documented in the DrugBank^23^ Drug Interaction Checker (OMx Personal Health Analytics Inc., Edmonton, AB, Canada) which is based essentially on these same three severity class definitions (https://dev.drugbank.com/guides/terms/severity). Of these ten top-ranking drug pairs, only five were assigned documented interactions in the DrugBank database. The severity of these interactions was Moderate for all but one pair consisting of dexamethasone and simvastatin. The latter was assigned a Major interaction severity, whereby concurrent administration of dexamethasone can increase elimination of simvastatin, decreasing serum concentrations and compromising its therapeutic effect^24^. Likewise, co-administering simvastatin with ruxolitinib (NCT04348695) can increase the latter’s serum concentration, affecting its tolerability and earning it a Major interaction assignment.

### Simulating proposed Interventions

**Fig. 6:**
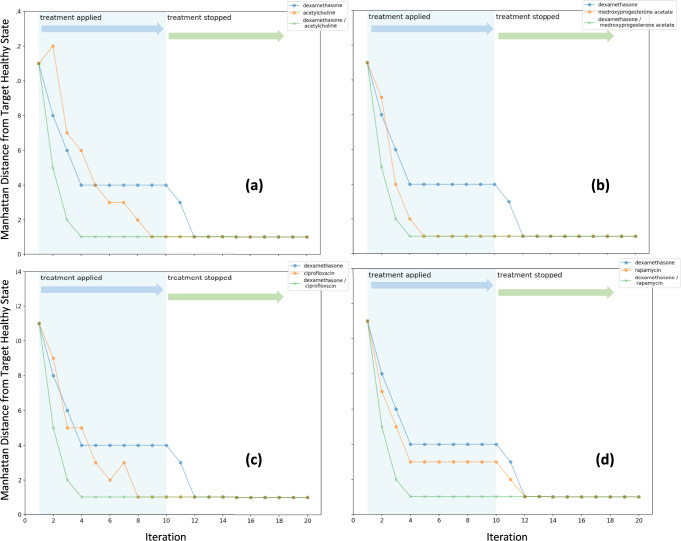
Simulating synergistic companions to dexamethasone. Discrete event simulation using synchronous state transition update of host immune network showing the overall Manhattan distance from the target immune resting state as a function of iteration. An idealized antiviral is applied to an initial persistent state approximating cytokine storm along with **a** dexamethasone alone, acetylcholine alone and a combination of both, **b** dexamethasone alone, medroxyprogesterone acetate alone and a combination of both, and **c** dexamethasone alone, ciprofloxacin alone and a combination of both, and **d** dexamethasone alone, rapamycin alone and a combination of both. In all cases, we see a positive synergy indicative of complementary drug actions.

**Fig. 7:**
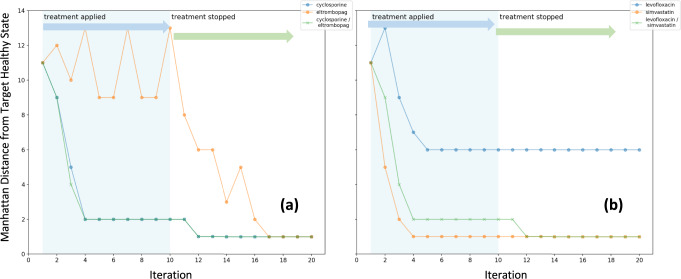
Simulating ineffective drug pairs. Discrete event simulation using synchronous state transition update of host immune network showing the overall Manhattan distance from the target immune resting state as a function of iteration. An idealized antiviral is applied to an initial persistent state approximating cytokine storm along with **a** cyclosporine alone, eltrombopag alone or a combination of both, and **b** simvastatin alone, levofloxacin alone or a combination of both.

It is important to recall that candidates from the initial set of 144 illness-relevant, pathway relevant, and model-relevant compounds were ranked on the basis of their respective ability to align with and support model-predicted and idealized sets of concurrent manipulations to specific immune mediator targets. As real-world drugs or combinations thereof may not necessarily align exactly with these idealized interventions, we conducted simulations to verify the suitability of the best-ranked candidates. Results presented in Figs. 6 and 7 show the gradual decrease in disparity (Manhattan distance) between the predicted immune marker co-expression profile at each iteration and the target signature at the desired immune resting state imparted by the simulated drug therapy and its eventual discontinuation. The initial state of the network is a persistent cytokine storm located at a Manhattan distance of 13 bits away from the immune resting state. Drugs actions are simulated in the context of a concurrently applied idealized antiviral resulting in the absence of a measurable viral load.

The simulated effects of administering dexamethasone in combination with the 4 companion drugs that provided the highest WM *E*_adj_ (Fig. 5), namely acetylcholine, medroxyprogesterone acetate, ciprofloxacin and rapamycin, are illustrated in Fig. 6. While all these drugs applied alone will eventually drive the immune network response to a stable recovery state, pairing dexamethasone with these companion drugs shows a noticeable positive synergy that in all cases delivers a much more expedient reduction in immune activation. Indeed, the predicted immune network response to all four drug pairs shows an immediate disruption of the cytokine storm state, rapidly achieving a residual distance from the immune resting state of only 1 bit within the first 4 iterations. This expediency is rivaled only by medroxyprogesterone acetate, with a repose time of 5 iterations, and is noticeably better than the responses of 8–12 iterations predicted for ciprofloxacin, acetylcholine and rapamycin. Importantly, all combinations produce a recovery that persists even after the intervention is discontinued. Different and more varied behaviors can be observed in drug pairs with decreasing values of WM *E*_adj_ that approach values obtained with single drugs (Fig. 5). For example, the pairing of cyclosporine with eltrombopag corresponds to an important reduction in WM *E*_adj_ or a loss in general applicability across multiple intervention paths that approaches that of a single drug. Accordingly, the simulated responses shown in Fig. 7a show a dominant effect of cyclosporine alone, with no additional benefit being contributed by the concurrent use of eltrombopag. At roughly the same reduction in WM *E*_adj_, but with a much higher maximum enrichment in individual intervention, the pairing of simvastatin with levofloxacin provide an even greater emphasis on narrow performance at the expense coverage across paths to favorable outcomes. This even narrower focus not only eliminates any benefits of pairing but leads to a negative synergy where the actions of simvastatin alone dominate and where the latter is only hampered by the addition of levofloxacin (Fig. 7b). These examples suggest that as WM *E*_adj_ decreases, any benefit of pairing two drugs also decreases with the addition of a second companion drug having no effect or even counteracting the actions of the dominant drug.

## Discussion

While computational approaches, many of which are rooted in molecular docking methodology^11^, have been proposed to accelerate the data-driven screening of compounds targeting the COVID-19 pathogen or host proteins recruited during infection, little attention has been directed towards the in silico design of pharmacologic strategies for mitigating excessive host immune response, a key contributor to the severity of COVID-related ARDS and associated morbidity^25^. Moreover, while important resources like the VirHostNet database document interactions between virus and host proteins^13^, the resulting interaction networks remain static representations of molecular compatibility where drug targets are inferred on the basis of network structure alone. Similar principles of similarity have also been applied to transcriptomic profiles to construct drug-disease networks though once again candidate compounds are identified on the basis of network structure alone^26,27^. Here, we use a previously reported causal model of host immune regulation constructed by our group from published prior knowledge^15^ to implicitly account for and exploit the dynamic propagation of such network effects, and the resulting regulatory stability of the system, to identify subsets of dynamically coupled drug targets^18^. We then systematically apply a number of quantitative criteria to rank a broad range of candidate FDA-approved drugs based on their ability to jointly manipulate these subsets of immune mediators such that their corresponding downstream regulatory interactions are optimally leveraged to effectively disrupt a persistent ARDS-induced cytokine storm. The regulatory stability of this immune hyper-responsive state^15^ was such that only by concurrently modulating at least four host immune mediators would the host immune response be robustly directed to stand down in the context of a reduced viral load. Indeed, a closer examination of the 16 most pharmaceutically accessible intervention strategies revealed that drugs offering a broader coverage, in particular as it relates to the suppression of CSF3, IFNL1, TNF and to a lesser extent that of IFNG and STAT1, typically ranked higher. For these reasons many highly anticipated corticosteroids and antibody drugs did now fare as well as expected. For example, the antibody drug infliximab did rank among the top 20 drugs (ranked #14) with 7 documented targets. However, only two of these seven actions (TNF and IFNG) involved the five highly represented targets mentioned above, assigning it a lower rank. Similarly, prednisolone (ranked #31) counted five documented targets that included three of the five preferred targets. However, one of these three targets (CSF3) was regulated in the opposite direction to the action prescribed by the intervention sets leading to a penalty in rank.

Out of 144 candidate drugs, even the 10 top-ranking candidates when used alone offered no better than a *P*_adj_ ~ 0.10 chance of enrichment above that of any single random immune modulator in directly harnessing at least one of the model-predicted optimal treatment mechanisms active in COVID-induced ARDS (p(Max *E*_adj_ = 0) ≥ 0.11). Not surprisingly, the actions of these individual drugs did not generalize well either. Indeed, the ability of any single drug to simultaneously recruit multiple alternate treatment pathways supporting the best outcomes was also highly variable, with none achieving significance over a random immune modulator (p_adj_(WM *E*_adj_) ≥ 0.14). Figuring prominently in this list are several drugs currently under active clinical investigation. Broadly studied in the general context of pulmonary distress, dexamethasone is currently the subject of at least 2 active Phase 3 clinical trials in COVID-induced ARDS specifically (NCT04836780; NCT04843761). Similarly, Phase 3 trials of the anesthetic sevoflurane, also studied in the broader context of ARDS, (NCT04355962; NCT04415060), the PPAR alpha agonist fenofibrate (NCT04661930), the calcineurin inhibitor cyclosporine (NCT04979884) and mTOR inhibitor rapamycin (Sirolimus)(NCT04948203) are currently active, with some recently completed. Also currently under active Phase 3 clinical study, we find acetylcholine maintenance or release by compounds such as nicotine (NCT04608201; NCT04598594) and pyridostigmine bromide (NCT04343963) ranking among the top-10 treatment strategies. In contrast, despite early interest in the antimalarials hydroxychloroquine and chloroquine, these aligned very poorly with our model-predicted intervention sets, ranking 66th and 96th out of the 144 candidate drugs. Consistent with this, subsequent data has not supported clinical effectiveness of the latter which together with broad off-target effects in the lung has led the US FDA to revoke Emergency Use Authorization (EUA) for CQ and HCQ as the known risks outweigh potential benefits of their use^28,29^.

As might be expected, we find both cyclosporine and rapamycin, known immune modulators commonly used to manage transplant rejection, ranking among the top five drugs in terms of their alignment with the best model-predicted interventions. The immunomodulatory, antiviral and tissue-protective properties of cyclosporine have recently been documented in the context of H1N1 and COVID-induced ARDS in several preliminary studies^30,31^; however, concerns remain regarding cyclosporine’s many side effects and the use of low-dose regimens are encouraged^32^. Similarly, though known to be an effective suppressor of Th17-mediated airway inflammation^33^ and T cell-driven lung injury^34^, investigation of rapamycin’s effectiveness and safety in treating COVID-induced ARDS remains in its early stages^35^. In sharp contrast with these well-known immune modulators, we also find drugs typically associated with the treatment of other conditions. For example, both simvastatin and fenofibrate are commonly used to treat dyslipidemia. Upregulation of the peroxisome proliferator-activated receptor-alpha (PPAR alpha) with compounds such as fenofibrate has been reported to also attenuate lung inflammation^36^ making it a candidate of legitimate interest in the treatment of COVID-induced ARDS and associated hypercoagulability^37^. However, results from a recent real-world observational study emphasize the need for rigorous randomized control trials before a clear benefit in a COVID setting can be confirmed^38^. Similarly, the anesthetic sevoflurane remains under active clinical study with limited evidence suggesting a contribution to host defenses engaging cytoprotective pathways around heme oxygenase-1 (HO-1) expression, a target of the SARS-cov-2 virus^39,40^. In a twist of irony, while smoking confers susceptibility to COVID-induced respiratory distress, there is mounting evidence that cholinergic agonists like nicotine^41,42^ and pyridostigmine bromide^43^ that promote acetylcholine expression not only offer significant anti-inflammatory actions^44,45^ but may also interfere with SARS‐CoV‐2 infection and viral replication.

Arguably, the best known of all these candidate drugs is the anti-inflammatory dexamethasone, with robust evidence supporting its efficacy and safety in the treatment of ARDS^46^. Indeed, recent molecular profiling has pointed to host immune response mechanisms dysregulated in COVID-induced ARDS that dexamethasone may be well-suited to modulate^3^. This is consistent with clinical observations of reduced all-cause mortality in cases of severe and critically severe ARDS reviewed in ref. ^47^, though these results appear to vary significantly based on subpopulation and timing of therapy^31^. However, evidence that its immunosuppressive effects lead to accelerated viral replication has fueled continued debate regarding its appropriate clinical use^48^. Some of these issues might be mitigated by combining dexamethasone with a companion drug in an effort to make it more transportable, more consistently robust, and potentially more efficient. Indeed, drug pairs were predicted in this work to perform substantially better in promoting the most robust and direct disruption of cytokine storm and to do so by concurrently harnessing multiple alternative pathways. Interestingly, dexamethasone was unanimously recruited into the top five pairs where it was combined with other high-ranking drugs. These imparted actions complementary to those of dexamethasone such as the downregulation of the immune activator CSF3. Acetylcholine agonists, medroxyprogesterone acetate, and ciprofloxacin ranked highest as companion drugs with identical intervention enrichment scores, followed by rapamycin and simvastatin. While acetylcholine agonists are under study, as noted above, this is less the case for medroxyprogesterone acetate and ciprofloxacin. Used in hormone replacement therapy and as a contraceptive, medroxyprogesterone acetate also exerts little-documented immune-modulatory actions, with high doses inhibiting the expression of inflammatory cytokines jointly affecting Th1, Th2, Th17, and Th22 immune signaling^21^, contributing to reductions in symptom severity and corticosteroid use in asthmatic subjects^49^. Fluoroquinolones such as the broad-spectrum antimicrobial ciprofloxacin, have been shown to also exhibit antiviral properties as well as significant immunomodulatory effects, including downregulation of IL-1 and TNF^50^ making these compounds of interest in the treatment of COVID infection and subsequent respiratory complications^51^. Similarly, clinical investigation of simvastatin in a COVID setting remains in its early stages. While inhibition by a statin of 3-hydroxy-3-methylglutarylcoenzyme A (HMG-CoA) reductase has been shown to interfere with the development of ARDS in an animal model^52^, clinical outcomes were initially found to be unchanged by simvastatin^53^. However, subsequent analysis of study data identified statin-responsive phenotypes^54^, prompting renewed interest in statins as modulators of thromboinflammatory pathways in COVID-induced ARDS^55,56^.

Unfortunately, while several of these top-ranking companion drugs are under independent study, none are yet being investigated in the context of a combination therapy. A possible exception to this would be the ongoing Phase 2 study pairing ruxolitinib with simvastatin (NCT04348695) which did not rank highly with the metrics used in this work. While this result in and of its own does not necessarily guarantee a poor outcome, it does suggest that other combinations might offer stronger synergies in leveraging multiple complementary drug-action pathways predicted to effectively disrupt ARDS cytokine storm. Indeed, the analysis presented in this work was never meant to replace more exhaustive simulation studies and experimental validation of drug responses. Nonetheless, this approach offers a computationally efficient quantitative framework for ranking individual drugs and drug combinations in a way that implicitly accounts for and exploits the network biology of interacting pathways using a mechanistic dynamic representation of broad community-wide peer-reviewed prior knowledge. While the metrics used here do not explicitly favor drug pairs expected a priori to deliver positive synergistic effects, it was interesting to observe that many such positive synergies seemed to emerge as a natural consequence of increased transportability across those multiple intervention paths predicted to produce better outcomes.

In this iteration, the proposed framework does not yet formally account for drug toxicity and known adverse events associated with drug combinations. Ongoing work is directed at including such penalties in the estimation of drug enrichment scores. In addition, this initial analysis does not explicitly include or leverage drug pharmacodynamic properties or drug dose-response data e.g., IC_50_ data, though such information is indirectly leveraged through literature-interpreted drug actions^57^. Where such data exist, the current framework could potentially benefit from concurrent use and validation against empirical drug data-driven approaches such as the Drug Atlas^58^ with the proviso that predictions made here include maximally synergistic drugs in addition to those that are maximally complementary in their actions. Similarly, recent digital repositories focused specifically on consolidating clinical experience and putative targets of COVID-19 therapeutics^59,60^ might also be leveraged to further inform the family of candidate drugs to be assessed in the context of host immune response dynamics captured here by our regulatory network model. Finally, in the current analysis, the selection of drugs useful in disrupting cytokine storm was limited to compounds having received FDA approval. The intention was to provide a sound basis for establishing credibility of the proposed drug selection method as more is typically known about the effects of such compounds. Moreover, repurposing of the most promising candidates might be facilitated by the fact that they are already in clinical use. Certainly, in future iterations of this analysis, experimental compounds would be included in the hope of further optimizing efficacy of treatment. Similarly, the optimal choice of targets and corresponding compounds might differ depending on the severity and phase of illness. While simulations of treatment response used here were conducted from an initial illness state corresponding to an established cytokine storm, this is not a requirement. Indeed, future work involving a broader search across a wider range of initial conditions might identify thresholds in severity where some drugs should be discontinued in favor of others.

Though opportunities exist for continued refinement of this approach, we contend that the current framework offers a first important step towards producing a reliable and computationally efficient means for the rapid repurposing of well-known compounds currently in clinical use and redirecting these towards the effective management of potentially severe comorbidities such as ARDS, thereby reducing fatalities from SARS-CoV-2 infection. This being said, it important to remember that the current model was adjusted to reproduce and capture host immune response to a SARS-CoV1 exposure, as this was the most readily available data at the time. Of course, new data sets continue to be shared that describe host responses to SARS-CoV2 specifically. Though we expect some degree of uniqueness in ARDS induced by this more recent pathogen, we believe that predictions presented here based on SARS-CoV1 would at the very least be relevant to the treatment of cytokine storm in SARS-CoV2 infection. This may be especially true given the coarser resolution afforded at the level of drug assignment. With these limitations in mind, we propose that the drugs and drug pairs identified nonetheless constitute a potentially promising set of initial candidates for further in silico, animal and clinical study.

## Methods

### A model of immune signaling in cytokine storm

As previously reported by our group^15^, a basic immune signaling network model was constructed from 19 molecular markers (nodes) with involvement in infectious pneumonia as documented in the Elsevier Knowledge Graph database^16^ (Elsevier, Amsterdam) using the Pathway Studio (Copyright © 2020 Elsevier Ltd. except content provided by third parties. Pathway Studio is a trademark of Elsevier Ltd.) suite of software tools^61^. This same database supported 112 regulatory interactions (edges) previously extracted from 2653 abstracts and/or full text of peer-reviewed journal publications using the MedScan natural language processing (NLP) engine^62,63^. The dynamic behavior of the network is governed by model parameter values that dictate the underlying transition from one network state to the next^64^ using an extension of discrete logic concepts put forward by Thomas and Thieffry^65,66^ and applied specifically to cell signaling systems by^67^ and others. More specifically, perception thresholds on incoming signals at each network node are used to mimic the actions of high and low-affinity receptors. Only those incoming signals that exceed their respective perception thresholds will be considered as active mediators of the target downstream node. Each possible combination of these active signals is assigned a respective logic weight that dictates the regulatory response at that node, i.e., increase, decrease or maintain the same expression level in the next iteration. In this way, each regulatory decision is context-specific, weighing the actions strong activators against those of weak inactivators and vice versa.

**Fig. 8:**
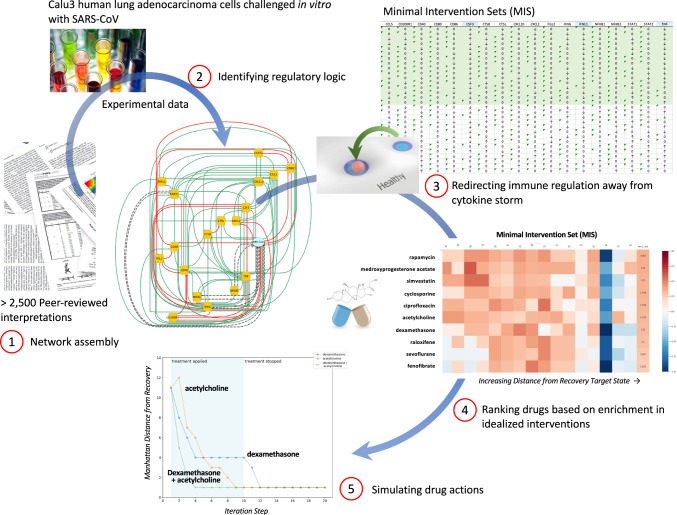
A regulatory network informed process for ranking candidate drugs. The computational workflow for identifying and ranking drugs available for repurposing to COVID-induced ARDS consists of several sequential steps, namely (1) a regulatory network is assembled that links the biological mediators of interest through documented regulatory interactions extracted by text-mining of the peer-reviewed literature, (2) sets of logical parameters capturing receptor affinity effects and context-specific regulatory responses are identified that support adherence to available data using a Constraint Satisfaction approach, (3) the resulting sets of competing dynamic network models are used to identify sets of target nodes that if manipulated concurrently would succeed in disrupting a persistent pathology like ARDS to restore normal immune regulation, (4) candidate compounds are then ranked based on how well and how specifically they support one or several of these idealized interventions. Finally, (5) the actions of top-ranking drugs and drug combinations are simulated to verify expected response dynamics.

Acceptable sets of these perception thresholds and decisional logic weights (*K*-values) were identified here using a constraint-based optimization framework^68^ such that the network’s corresponding dynamic behaviors encompassed data describing the 72-h in vitro time course response of human Calu-3 cells to SARS-CoV infection^69^. Given the still limited availability of molecular data specific to COVID-19 infection and the similarity in clinical presentation to SARS^70^, we consider this an acceptable proxy for this exercise. These data were retrieved from the Gene Expression Omnibus (GEO accession number GSE33267) and normalized to the mean of the mock infection samples (control) at each timepoint and translated into discrete expression levels using Variational Bayesian Gaussian clustering^71^. As the complexity of the model exceeds that of the available experimental data, the parameter identification problem is said to be underdetermined. In this case, 19 unique models were identified with <5% departure from the experimental data, with 11 of these parameter sets explaining the time course data exactly (0% error). Of these, 3 parameter sets (models 15, 17, and 18) supported steady-state expression profiles that closely approximated an immune resting state and one considered representative of the persistent cytokine storm. In the case of one model in particular, the basin of attraction surrounding the inactive immune resting state conferred a sufficiently robust response to infection for cytokine storm to occur at a clinically realistic rate^8^ (~15%;) (26% predicted by model 18). As such, this model was retained as the basis for all simulated drug actions presented here. Additional details may be found in a concurrent report by our group^17^. These steps and the proposed drug selection process are illustrated as a workflow in Fig. 8.

### Minimal intervention set identification

As mentioned in the previous section, we use a discrete decisional logic to manage the flow of immune signals through the host regulatory network and direct its evolution from one immune state to the next. At any given iteration, each of the network’s immune mediator nodes is assessed for incoming signals expressed above their respective perception thresholds. As mentioned above, based on the specific combination of active upstream mediators and their mode of action (activator or inactivator), the state of a corresponding downstream node is predicted to either remained unchanged, increase or decrease in the next time step. Applying this to all nodes, the next state towards which the overall network should progress given the current state, namely the network image, is computed. According to the update scheme being used, this predicted change in state is applied to a single random node (asynchronous update) or to all eligible nodes simultaneously (synchronous update). As we were primarily interested in stable persistent behaviors like cytokine storm, simulations of immune response were conducted here using a synchronous update of all node states for reasons of computational efficiency. Using this framework, model predictions of host inflammatory response under different conditions were conducted as part of a multi-objective optimization problem directed at identifying the smallest or minimal sets of molecular targets in the immune network that when manipulated concurrently in a specific manner (i.e., upregulated or downregulated) would theoretically disrupt a fully established cytokine storm and return immune activation to a normal resting state either fully or partially. Here we describe a fully established cytokine storm as the co-expression pattern of immune mediators measured experimentally in the Calu-3 cell cultures 72-h after coronavirus infection^69^ (GEO accession number GSE33267). In addition, the baseline healthy resting state was specified as an idealized immune quiescent state where all immune mediator entities are expressed at a nominal activation level (e.g., inactive or 0 in the discrete space).

These Minimal Intervention Sets (MIS) were identified by solving a computationally efficient Constraint Satisfaction Problem (CSP)^19^ where the idealized manipulations of at most nine molecular targets at a time, or 50% of the network, were iteratively assessed. As reported in previous work by our group^18^, this optimization consisted of concurrently minimizing the number of immune nodes being targeted, the final distance to the target state achieved by the intervention (calculated using the L1-norm), and the number of state transitions required (efficiency) to reach this treatment stabilized state. This multi-objective optimization was stated as a constraint satisfaction problem (CSP)^72^ in MiniZinc^73^ and solved with the greedy solver Chuffed^74^.

### Ranking intervention sets as candidates for repurposing

By design, solving a constraint satisfaction problem allows for the recovery of all solutions which comply with the constraints stated. As mentioned above, these constraint-compliant solutions can be ranked further based on other performance metrics such as size or cardinality of the intervention target set, as well as the predicted expediency (efficiency) of response and reliability of outcome (robustness) as reported previously by our group^18^. However, despite their optimality in achieving a desired outcome, such idealized interventions (concurrent up or downregulation of specific targets) may not translate readily into clinical care using currently available drugs. Here, we propose a quantitative metric to assess the actionability *A* of an idealized MIS solution as a ready candidate for drug repurposing^75^. The *A* metric is composed of four component scores. The first of these, cardinality, is the number of prescribed targets in the MIS vector *m*.

1 $$C(m) = \left\| m \right\|_0$$

A second metric is the antagonist ratio. Because it is generally more common for drugs to exhibit inhibitory actions against molecular targets, MIS solutions with a higher ratio of antagonist-to-agonist actions are prioritized.

2 $$V\left( m \right) = \frac{p}{q}\left\{{\begin{array}{*{20}{l}} {p = \mathop {\sum}\nolimits_{i = 1}^n {[m_i = 1]} } \\ {q = \mathop {\sum}\nolimits_{i = 1}^n {[m_i = - 1]} } \end{array}} \right.$$

Where *n* is the number of targets in *m, m*_i_ is the polarity of the target at index *i, −*1 represents an inhibition of the target, and +1 represents activation of the target.

A third consideration is a broad accessibility to equivalent pharmacotherapy, expressed here as the number of alternate drugs available to perform the same actions specified in a given MIS solution.

3 $$R\left( m \right) = \left( {\mathop {\prod}\nolimits_{i = 1}^n {\hat m_i} } \right)^{1/n}$$

Where *n* is the number of targets in *m* and $\hat m$ is the set of counts of all drugs that act with correct polarity on each element of *m*.

Finally, we attempt to account for off-target effects of all drugs applicable to an MIS, that is all drug actions inadvertently applied outside of those dictated in the MIS. Accordingly, the actionability of an MIS is penalized or discounted in proportion to the minimum number of documented off-target interactions, affected by all drugs at each target and cumulated across all component targets in the MIS (Eq. (4)).

4 $$T\left( m \right) = \left( {\mathop {\prod}\nolimits_{i = 1}^n {\min \left( {k_i} \right)} } \right)^{1/n}$$

Where *n* is the number of targets in *m* and *k* is a vector of *n* elements, each of which is itself a vector of length *j*. Each *k*_*i,j*_ is the total number of interactions known for the drug at *j*. To be included in *k*_*i*_*, j* must interact with *m*_*i*_ with the correct polarity.

These components are combined into a single aggregate score as shown in Eq. (5). Two tuning parameters, γ and τ, are used to adjust the relative weight of solution cardinality and the antagonist ratio, respectively. For this analysis, γ and τ were set to 1.0 to assign equal weight to all component scores. Of note, any MIS solution where the drug-action databases do not contain any drug known to interact with one or more targets is considered an unactionable solution or one that is not suitable for repurposing.

5 $$A\left( m \right) = \frac{{R\left( m \right) \ast V\left( m \right)^\tau }}{{C\left( m \right)^\gamma \ast T(m)}}$$

The statistical significance of an actionability score was estimated empirically from a null distribution of actionability for randomly generated mock MIS solutions. The null distribution was generated by repeatedly sampling a discrete uniform distribution along the integer interval [−1, 1], where each mock MIS solution vector is equal in length to the maximum possible cardinality allowed for in the current model (i.e., the number of allowable targets ≤ number of network nodes). The actionability scores corresponding to these artificial solution sets form the null distribution. Empirical *P* values for each real solution are then estimated as the proportion of artificial actionability scores greater than or equal to the actionability of each real solution. To account for multiple tests, the Benjamini–Hochberg^76^ correction is applied.

6 $$\hat p = \frac{{(r + 1)}}{{(n + 1)}}$$

Where *n* is the number of simulated solutions and *r* is the number of simulated solutions whose actionability is greater than or equal to the actionability of the actual solution. All actionability scores and null *P* values were computed using Python version 3.8.3 (2020–05–13) (https://www.python.org/).

### Creating a pool of problem-relevant candidate drugs

A broad preliminary set of candidate drugs with broad relevance to the problem of interest was assembled by applying three complementary surveys of the Elsevier (Amsterdam, NL) Knowledge Graph database^16^ (Fig. 2a). The first and perhaps broadest of these consisted of searching for immune modulators associated with at least one node in the network model developed by our group^15^, yielding 5120 drugs. A second more infection-focused survey included all FDA-regulated compounds known to act as agonists or antagonists of human proteins and protein families found characteristic of Coronavirus infection, Severe Acute Respiratory Syndrome Coronavirus 1 (SARS-Cov-1) and 2, (SARS-Cov-2; COVID-19) as well as the Middle East Respiratory Syndrome coronavirus (MERS-CoV), producing a set of 1211 drugs. A third more clinically motivated survey focused on drugs with targets implicated in the pathogenesis and progression of coronavirus-induced ARDS. This third set consisted of 1270 candidate drugs known as (a) affecting autophagy, or (b) being the object of past or ongoing clinical research for the treatment of SARS, MERS, or COVID-19. The intersection of individual results from each of these three complementary surveys, produced a consensus list of 144 FDA-regulated compounds, drawn from 94 Established Pharmacologic Classes (EPC), that were relevant to the pathogen, to the progression of infection and to host immune response in ARDS-induced cytokine storm. Of these, 67 are registered to interventional clinical trials in the United States as of July 27, 2020 (Fig. 2b). This set of drugs includes monoclonal antibodies, non-steroidal anti-inflammatories, antibiotics, statins, and other immunomodulatory compounds but the most widely represented are kinase inhibitors and corticosteroids (Fig. 2c).

Of note, in estimating the statistical significance for the enrichment of a drug *E*_adj_ with respect to a specific intervention solution (MIS) we compare against enrichment E0_adj_ obtained in a background set of drugs serving as a null distribution. In order to avoid including an overwhelming majority of drugs for which no targets exist in the model, introducing a bias of the null distribution towards no enrichment, we define a background set consisting of only those 1900 drugs which like the candidate drugs (foreground) not only show affinity for at least one entity in the network but also modulate this target according to a well-defined mode of action, namely a specific upregulation or downregulation of the target by the drug. In the case of drug pairs, we establish a similar null distribution by randomly subsampling 50,000 instances from the over 1.8 million possible combinations of two drugs selected from these same 1900 candidates. In both cases, these sets of model-relevant drugs remain broad while also offering more conservative measures of the significance threshold for enrichment E0_adj_.

### Ranking drugs that optimally support an intervention

To facilitate the comparison of individual drugs or drug combinations that might support a specific model-predicted intervention to a greater or lesser extent, we propose as a quantitative ranking metric, the Adjusted Intervention Enrichment Score (*E*_adj_). The *E*_adj_ score is a weighted measure of the degree to which set of drug actions enriches an MIS solution. First, a basic enrichment *E* is defined as the percentage of prescribed targets in the MIS solution that are acted upon by a given drug or combination of drugs. However, this basic measure of coverage does not account for an “overenrichment”, in which an entity unprescribed in the MIS solution is modulated by a candidate drug, nor does it account for conflicting modes of drug action, where a candidate drug acts on one or more targets in a manner contrary to that prescribed in the MIS solution. To account for such undesirable occurrences, two penalty scores, the overenrichment penalty, *b*, and the conflict penalty, *c*, are used to adjust the raw E score. It is this adjusted E score, *E*_adj_, that is used to compare competing drugs and drug combinations.

Consider an MIS solution to be a vector, *m*, whose length is equal to the number of entities or nodes in a model and whose elements *m*_*i*_ can hold one of three possible values^18^, namely −1, 0, or 1. A value of *m*_*i*_ = 0 indicates no action on the corresponding model entity, a value of −1 indicates a required downregulation or inhibition, and 1 indicates a required upregulation or activation of that entity or node respectively. Similarly, the actions of a drug, *d*, can be represented by a vector of the same length as *m* and whose elements can hold the same value, but where those values are determined by known interactions between the drug and the elements of the corresponding model. The basic enrichment score *E*(*d*,*m*) is therefore the number of elements in *d* whose values are equal to their corresponding elements in *m* divided by the number of nonzero elements in *m*. The following equations, use Iverson Bracket Notation^77^ where statements in square brackets evaluate to a Boolean state of 1 if true and 0 otherwise.

Enrichment *E*(*d*,*m*) is defined as:

7 $$E(d,m) = \frac{{\mathop {\sum }\nolimits_{i = 1}^n \left[ {\left( {d = m_i} \right)\wedge{\left( {m_i \,\ne\, 0} \right)}} \right]}}{{\mathop {\sum }\nolimits_{i = 1}^n \left[ {\left( {m_i\, \ne \,0} \right)} \right]}}$$

where *n* is the number of elements in *m*. To account for overenrichment or direct off-target effects, a penalty, B is subtracted from the raw *E* score.

8 $$B(d,m) = \frac{{\mathop {\sum }\nolimits_{i = 1}^n \left[ {\left( {d_i \,\ne \,m_i} \right)\wedge{(m_i = 0)}} \right]}}{n} \cdot b_0$$

where *b*_*0*_ is a user-defined penalty weight. Larger values of *b*_*0*_ penalize more greatly for overenrichment. Another penalty accounting for contrary drug actions, *C* is also subtracted from the raw *E* score to account for conflicting actions on a target.

9 $$C(d,m) = \frac{{\mathop {\sum }\nolimits_{i = 1}^n \left[ {\left( {d_i\, \ne \, m_i} \right)\wedge{\left( {m_i \,\ne\, 0} \right)}} \right]}}{n} \cdot c_0$$

where *c*_*0*_ is a user-defined penalty weight. Larger values of *c*_*0*_ penalize more greatly for conflicting actions. Subtracting *B* and *C* from *E* yields the adjusted enrichment score, *E*_adj_.

10 $$E_{adj}(d,m) = E(d,m) - B(d,m) - C(d,m)$$

Here, we estimate the statistical significance of an adjusted enrichment score *E*_adj_ for a given drug or combination of drugs with respect to a specific MIS solution by comparing it against the distribution of the enrichment scores E0_adj_ obtained by randomly sampling drugs or combinations of drugs from a background set of 1900 drugs that exert a well-defined action on at least one network element, as described in the previous section. Empirical *P* values are estimated as the proportion of E0_adj_ scores greater than or equal to each observed *E*_adj_ score. These raw *P* values are then corrected for multiple comparisons across the subset of 144 drugs of interest (both model and illness-relevant) using Benjamini–Hochberg^76^. This process is analogous in some ways to gene set enrichment analysis (GSEA)^78^, though the definition of enrichment and exact statistical methods differ.

To provide a measure of overall performance, the weighted percentile median enrichment^79^ is calculated for each drug or drug combination across all idealized intervention sets found to be significantly actionable (see the previous section “Ranking intervention sets as candidates for repurposing”). Here, the weight applied to each individual MIS solution is computed as the inverse square of the Manhattan distance separating the simulated treatment endpoint and the desired target state, such that MIS solutions which achieve better proximity to recovery from ARDS are weighted more heavily. This method produces the *E*_adj_ corresponding to the median rank MIS solution adjusted for quality of outcome. The median absolute deviation of the *E*_adj_ values about this weighted percentile median score is also computed to describe variability in *E*_adj_ across the MIS solutions. The significance of this weighted median performance *P*_adj_(WM *E*_adj_), as well as for the maximum enrichment *P*_adj_(Max *E*_adj_), both adjusted for multiple comparisons, are obtained by comparing against the same metrics computed for drugs and drug combinations selected randomly from the background set.

### Simulation of drug interventions

The response of the model immune signaling network^15^ to the actions of a drug or drug combination can be simulated as a sequence of expression profiles or network states that emerge as a result of the direction and type of interactions acting at each node as well as the parameters defining the underlying state transition logic^64^. These logic parameters mimic the effects of receptor affinity as well as the context-specific nature of immune response. Given its current state and a combination of incoming signals that meet or exceed their individual thresholds of action, the state of a given node at the next time step is set to the entry in that node’s truth table that corresponds to a response that might be expected under the same combination of upstream stimuli given the available experimental data^68^. As a synchronous update scheme was used here, these corresponding next states are applied to all nodes across the network simultaneously. However, this is not a limitation of the method and other update schemes can be used, including fully asynchronous update as well as a novel priority class update with memory^64,80^.

Accordingly, the dynamic response of the network to an external perturbation, such as a drug, can be simulated if the targeted network entities and the way in which they are modulated by the drug are known (i.e., promoting or inhibiting). When a drug targets an entity with a positive mode of action, the state value of that entity will increase incrementally until the maximum value for that entity is reached. The reverse holds true for a drug that exerts a negative action on its targets. For example, let A be an immune network entity with an allowable range of activation between 0 and 4, and where its current activation level is 2. When an agonist that directly targets A is applied, the activation level of A will increase from 2 to 3 in one iteration, and then from 3 to 4 in the following iteration, and will remain at 4 as long as the intervention is applied.

## Supplementary information

Supplementary Information

## Data Availability

Data files are available in Supplementary Information (Additional File 1) and on the GitHub repository: https://github.com/rghccsb/old-drugs-new-tricks.

## Code availability

Prototype tools and models developed specifically within the scope of this work are freely available to the community upon request for non-commercial use and research purposes only. These are available under a set of standard licensing terms protecting ownership by the Center and legislating not-for-profit use responsibilities and liabilities. These files may be found on the GitHub repository: https://github.com/rghccsb/old-drugs-new-tricks.
